# Selection and Validation of Candidate Reference Genes for Gene Expression Analysis by RT-qPCR in *Rubus*

**DOI:** 10.3390/ijms221910533

**Published:** 2021-09-29

**Authors:** Yaqiong Wu, Chunhong Zhang, Haiyan Yang, Lianfei Lyu, Weilin Li, Wenlong Wu

**Affiliations:** 1 Jiangsu Key Laboratory for the Research and Utilization of Plant Resources, Institute of Botany, Jiangsu Province and Chinese Academy of Sciences, The Jiangsu Provincial Platform for Conservation and Utilization of Agricultural Germplasm, Qian Hu Hou Cun No. 1, Nanjing 210014, China; ya_qiong@126.com (Y.W.); chzhang0714@163.com (C.Z.); haiyanyang_025@126.com (H.Y.); njbglq@163.com (L.L.); 2 Co-Innovation Center for Sustainable Forestry in Southern China, College of Forestry, Nanjing Forestry University, 159 Longpan Road, Nanjing 210037, China

**Keywords:** expression stability, blackberry, raspberry, reference gene

## Abstract

Due to the lack of effective and stable reference genes, studies on functional genes in *Rubus*, a genus of economically important small berry crops, have been greatly limited. To select the best internal reference genes of different types, we selected four representative cultivars of blackberry and raspberry (red raspberry, yellow raspberry, and black raspberry) as the research material and used RT-qPCR technology combined with three internal stability analysis software programs (geNorm, NormFinder, and BestKeeper) to analyze 12 candidate reference genes for the stability of their expression. The number of most suitable internal reference genes for different cultivars, tissues, and fruit developmental stages of *Rubus* was calculated by geNorm software to be two. Based on the results obtained with the three software programs, the most stable genes in the different cultivars were *RuEEF1A* and *Ru18S*. Finally, to validate the reliability of selected reference genes, the expression pattern of the *RuCYP73A* gene was analyzed, and the results highlighted the importance of appropriate reference gene selection. *RuEEF1A* and *Ru18S* were screened as reference genes for their relatively stable expression, providing a reference for the further study of key functional genes in blackberry and raspberry and an effective tool for the analysis of differential gene expression.

## 1. Introduction

Blackberry and raspberry are important members of the genus *Rubus*, which belongs to Rosaceae, a moderately large family, with an estimated 85 genera and more than 2000 sexual species [1]. At present, there are many excellent cultivars of blackberry and raspberry worldwide with different fruit colors, such as yellow, red, and black. Blackberry and raspberry fruits are rich in anthocyanins, organic acids, dietary fiber, vitamins, and minerals [1,2]. They also have antioxidant, anticancer, and anti-cardiovascular medicinal properties [3]. With increasing attention given to healthy diets, blackberry and raspberry fruits have shown broad developmental prospects because of their unique flavor and medicinal effects [4,5].

Quantitative real-time fluorescent polymerase chain reaction (RT-qPCR) is a commonly used method for the determination of gene expression and has been widely used in disease diagnosis and detection, drug development, and scientific research [6,7,8,9]. This method has many advantages, such as quantitative accuracy, repeatability, and high sensitivity [7,9]. When RT-qPCR is used to calculate the relative expression level of target genes (data standardization processing), it is necessary to combine relatively stable reference genes for correction and homogenization to improve the accuracy of quantitative results [8,10]. The commonly used reference genes are usually stable housekeeping genes, which are expressed in all kinds of cells and code for proteins necessary to maintain the basic biological activities of cells, for example, the *18S* gene, the most abundant ribosomal RNA in eukaryotic organisms, EEF1A and EEF1B proteins of the *elongation factor 1* (*EEF1*) gene family in eukaryotes, *polyubiquitin* (*UBC*), *ubiquitin* (*UBQ*), *F-box family protein* (*F-box*) and *actin* (*ACT*) [11,12]. Ideal reference genes require relatively constant expression under different treatments, in different tissues or organs, and at different developmental stages of cells. However, studies have shown that in actual experiments, no reference genes can be expressed stably under all conditions [7,8,13]. If unscreened reference genes are used, experimental data will be biased, and the reliability of target gene expression results will be affected. Therefore, reference genes with relatively stable expression should be screened according to specific experimental materials and conditions.

In recent years, studies on internal reference gene screening for different species, cultivars, and tissues have been developed. The most appropriate internal reference genes have been selected from fruit plants such as strawberry [6], blueberry [8], and pear [9] for mechanistic studies of growth and development, fruit ripening, and stress responses. However, there is no report on internal reference gene screening related to blackberry and raspberry. With the development of transcriptome sequencing technology, genes related to anthocyanin synthesis, hormone signal transduction, and metabolic pathways in *Rubus* plants have been mined based on transcriptome data. None of the above studies carried out the functional verification of related genes among different cultivars. Therefore, in this study, based on blackberry transcriptome sequencing data, predicted coding DNA sequence (CDS) data, and other information, 12 genes were chosen as candidate reference genes. The RT-qPCR technique was used to detect the expression level of candidate internal reference genes in different organs and fruit different development stages of *Rubus*. Then, geNorm, NormFinder, and BestKeeper software programs were used for a comprehensive analysis of the expression stability of the candidate genes. The aims of this study were to (i) evaluate the expression stability of 12 candidate reference genes, and (ii) screen/select the most stable internal reference genes expressed in different cultivars of *Rubus* (blackberry and raspberry), and different tissues/organs and fruit development stages, to provide the reference basis for the further study of fruit development and key functional genes in blackberry and raspberry.

## 2. Results

### 2.1. Selection and Expression Levels of Reference Genes

**Figure 1:**
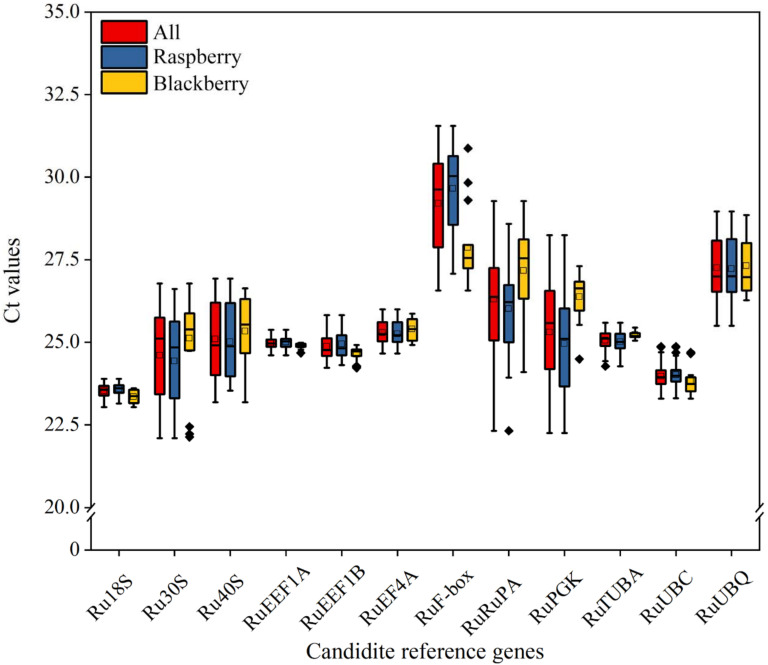
Cycle threshold (Ct) values of 12 candidate reference genes in all 72 samples, blackberry and raspberry. Ct distribution is presented as a box-plot, indicating the 25th and 75th percentiles. Lines across the boxes represent the median. The whisker caps indicate the maximum and minimum values. The dots represent the outlier.

In this study, 12 genes (*Ru18S*, *Ru30S*, *Ru40S*, *RuTUBA*, *RuEEF1A*, *RuEEF1B*, *RuEF4A*, *RuF-box*, *RuUBC*, *RuUBQ*, *RuPA*, and *RuPGK*) were selected as candidate reference genes. The primer annealing temperature was between 60 °C and 61 °C, and the amplification product length was between 72 bp (*RuF-box*) and 193 bp (*RuEEF1B*) (Table 1). The green fruits, immature fruits and mature fruits, leaves, stems, and stem apexes of raspberry and blackberry were used as templates to amplify different candidate reference genes. The specificity of the primers was evaluated by the RT-qPCR solution curve, and the results showed that all the target reference genes had a specific single peak; that is, all the primers had good specificity and could be used for the RT-qPCR analysis of gene expression. The expression level analysis of 12 candidate reference genes showed significant differences in cycle threshold (Ct) values among all 72 samples, and there were also differences in Ct values of internal reference genes between different cultivars of raspberry and blackberry (Figure 1). For all samples, the Ct values ranged from 22.101 to 31.549, with a mean of 25.453. The ranges of *RuEEF1A* (24.607–25.381) and *Ru18S* (23.037–23.893) gene expressions were smallest, and the ranges of *RuPA* (22.322–29.277) and *RuPGK* (22.249–28.238) gene expressions were largest.

### 2.2. Estimation of Stability by geNorm Analysis

**Figure 2:**
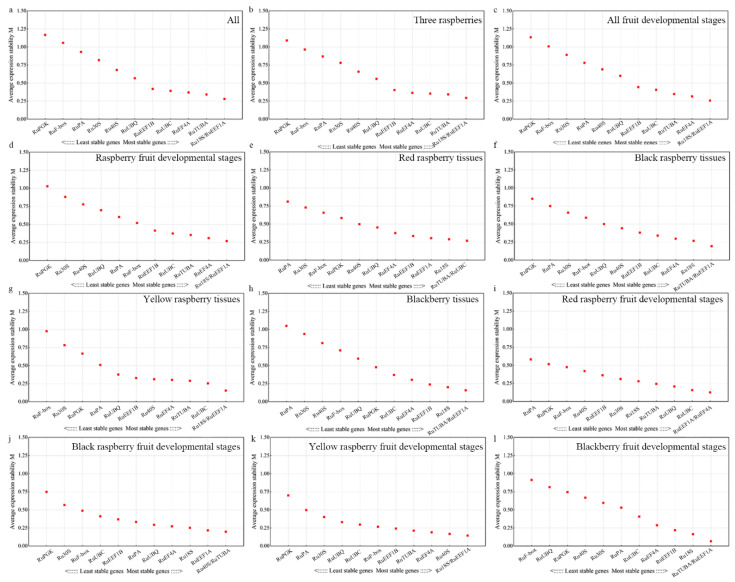
The average expression stability (M) values of the 12 candidate reference genes using geNorm analysis. Results from (**a**) All 72 samples; (**b**) Three raspberries; (**c**) All fruit developmental stages; (**d**) Raspberry fruit developmental stages; (**e**) Red raspberry tissues; (**f**) Black raspberry tissues; (**g**) Yellow raspberry tissues; (**h**) Blackberry tissues; (**i**) Red raspberry fruit developmental stages; (**j**) Black raspberry fruit developmental stages; (**k**) Yellow raspberry fruit developmental stages; (**l**) Blackberry fruit developmental stages. The least stable genes are listed on the left, while the most stable genes are listed on the right.

The geNorm software algorithm uses the mean expression stability measurement (M) value and paired variation (V) value of standardized factors to determine the stability of candidate reference genes and the number of suitable internal reference genes. An M value less than 1.5 indicates that the gene is suitable for internal reference; otherwise, it is not suitable for internal reference. The lower the value of M, the more stable the expression of reference genes. In all 72 samples, *Ru18S* and *RuEEF1A* were the most stable reference genes, followed by *RuTUBA* and *RuEF4A* (Figure 2a). Moreover, *Ru18S* and *RuEEF1A* were the most stable genes among the three raspberry cultivars, all fruit developmental stages, raspberry fruit developmental stages, yellow raspberry tissues, and fruit developmental stages (Figure 2b–d,g,k). *RuTUBA* and *RuEEF1A* were the most stable in the blackberry tissues and fruit developmental stages (Figure 2h,l). The most stable reference genes in the black raspberry tissues, red raspberry tissues, and red raspberry fruit developmental stages were *RuTUBA/RuUBC, RuTUBA/RuEEF1A*, and *RuEEF1A/RuEF4A* (Figure 2e,f,i), respectively. Interestingly, the M values of all candidate reference genes in black raspberry and red raspberry were less than 1.25.

**Figure 3:**
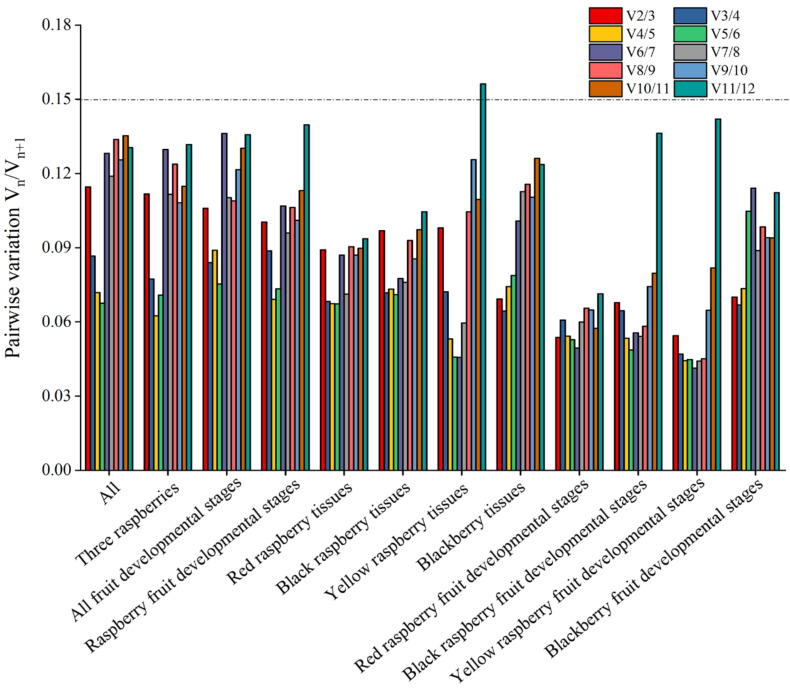
geNorm analysis of paired variation V values of 12 candidate reference genes. Vn/Vn + 1 values are used to determine the optimal number of reference genes. The cut-off value to determine the optimal number of reference genes for RT-qPCR normalization is 0.15.

In addition, the paired variation V value of the normalization factor after the introduction of a new gene can also be calculated in the geNorm program, and the optimal number of internal parameters can be determined according to the value of Vn/Vn + 1. The ratio of V2/3 in all samples was less than the recommended value of 0.15 (Figure 3), indicating that the number of internal reference genes used in the comprehensive analysis of all samples should be at least two. In other experimental groups, V2/3 was also less than 0.15, indicating that the optimal number of reference genes to use was two (Figure 3).

### 2.3. Estimation of Stability by NormFinder

**Figure 4:**
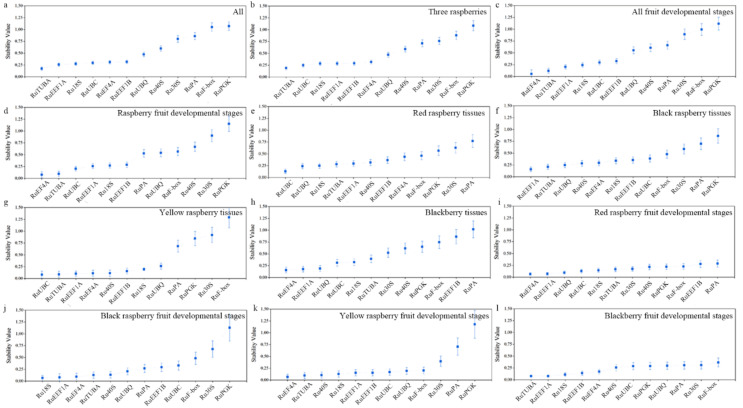
Gene expression stability of 12 candidate reference genes using NormFinder analysis. The lower the stability value, the more stable the expression level. (**a**) All 72 samples; (**b**) Three raspberries; (**c**) All fruit developmental stages; (**d**) Raspberry fruit developmental stages; (**e**) Red raspberry tissues; (**f**) Black raspberry tissues; (**g**) Yellow raspberry tissues; (**h**) Blackberry tissues; (**i**) Red raspberry fruit developmental stages; (**j**) Black raspberry fruit developmental stages; (**k**) Yellow raspberry fruit developmental stages; (**l**) Blackberry fruit developmental stages.

Similar to geNorm, the stability of each candidate reference gene in NormFinder software is also based on the stability (S) value of candidate reference genes. The lower the S value is, the higher the stability is. In all samples and across all three raspberry and blackberry fruit developmental stages, the candidate *Ru18S* reference gene was the most stable (Figure 4a,b,i). Among all (raspberry and blackberry) fruit, three raspberries, red raspberry and yellow raspberry fruit developmental stages, the *RuEF4A* gene was the most stable reference gene (Figure 4c,d,i,k). In the red raspberry tissues and yellow raspberry tissues, the *RuUBC* gene was the most stable reference gene (Figure 4e,g). In the black raspberry tissues and blackberry tissues, the most stable internal reference gene was *RuEEF1A* (Figure 4f,h), while in the black raspberry fruit developmental stages, the stability of the internal reference genes was as follows: *Ru18S* > *RuEEF1A* > *RuEF4A* (Figure 4j). In the comprehensive analysis, *RuPGK* showed the lowest stability, accounting for 7/12 (Figure 4).

### 2.4. Estimation of Stability by BestKeeper

According to the Ct value of each gene, BestKeeper software directly calculates three variables: the coefficient of variation (CV), standard deviation (SD), and correlation coefficient. In general, stable reference genes have a small SD value, and genes with an SD value greater than 1 are considered unstable genes. The SD value of the *RuEEF1A* gene among all samples, three raspberries, and all fruit developmental stages, yellow raspberry tissues, blackberry tissues, and blackberry fruit developmental stages, was lowest, so the *RuEEF1A* reference gene was the most stable among the six groups (Table 2). The stability of the *Ru18S* gene was highest in four groups (raspberry fruit developmental stages, red raspberry tissues, red raspberry fruit developmental stages, and yellow raspberry fruit developmental stages). In addition, in the black raspberry tissues and black raspberry fruit developmental stages, the stability of the three most stable potential reference genes was in the order *RuTUBA* > *Ru18S* > *RuEEF1A* (Table 2).

### 2.5. Comprehensive Ranking Analysis

Due to the different principles of the three statistical algorithms (geNorm, NormFinder, and BestKeeper), the results for the most stable reference genes were not completely consistent. To obtain more accurate and reliable analysis results, it is necessary to jointly analyze the ranking results of the three algorithms. Hence, the sum of the gene stability rankings with the three algorithms was used as a new variable, representing the comprehensive evaluation of gene expression stability in this study (Table 3). The smaller the sequence value, the higher the stability of gene expression. The comprehensive analysis showed that the *RuEEF1A* and *Ru18S* genes were the most stable internal reference genes among all the samples for the three raspberries, all fruit developmental stages, and yellow raspberry tissues (Table 3). Except for the stability of the *Ru18S* gene in the fruit developmental stages of red raspberry, in which it ranked fourth, in all other samples, it ranked in the top three.

The *RuEEF1A* reference gene was the fourth most stable in red raspberry tissue, while in the other groups, it was in the top three. In short, *RuPGK* was the least stable reference gene, and *RuEEF1A* and *Ru18S* were the most stable reference gene combinations in all sample analyses.

### 2.6. Validation of the Selected Candidate Reference Genes

**Figure 5:**
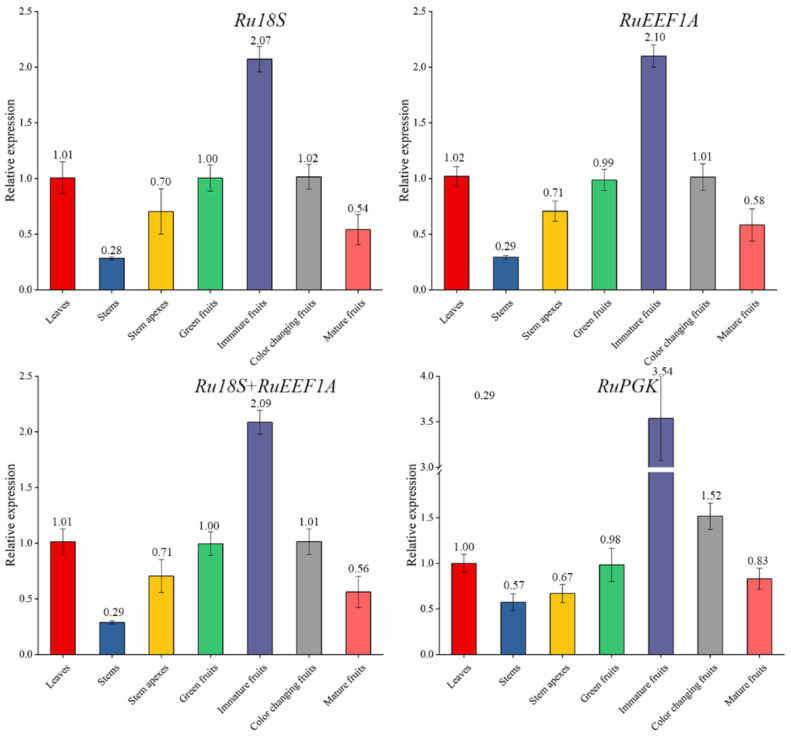
Expression patterns of RuCYP73A normalized to the top two stable genes (*Ru18S* and *RuEEF1A*) and an unstable gene (*RuPGK*).

According to the transcriptome data of blackberry fruit maturation, we found that the *RuCYP73A* enzyme gene in the flavonoid biosynthesis pathway was significantly differentially expressed among the fruit developmental stages. Moreover, through our preliminary research analysis, we found that the expression of *RuCYP73A* was also different in different organs or fruit development stages. Based on this, to validate the reliability of the stable reference genes, the relative expression patterns of the *RuCYP73A* gene were examined using different combinations of reference genes in different tissues and fruit developmental stages of blackberry. The two most stable reference genes (*RuEEF1A* and *Ru18S*) and the least stable reference gene (*RuPGK*) selected from the analyses described above were used either alone or in combination for RT-qPCR analyses. Although the overall relative expression patterns of the *RuCYP73A* gene showed similar trends, a difference was observed when the data were normalized to those of the different reference genes (Figure 5). Similar expression patterns were observed when we used the single genes as reference genes. However, when the least stable gene (*RuPGK*) was used as the reference gene, the expression levels of *RuCYP73A* showed significant fluctuations, and the expression patterns of green fruits and fruits with changing colors were not consistent with those observed when more appropriate reference genes were used (Figure 5).

## 3. Discussion

In recent years, with molecular biology methods being applied to various research fields, the study of gene expression and regulatory mechanisms has become a hot spot. RT-qPCR technology has the advantages of high precision, throughput, and sensitivity, and has become an important tool for molecular biology research. The analysis of gene expression by RT-qPCR is an important method commonly used to understand biological regulatory mechanisms [14]. Due to the accuracy of RT-qPCR results being easily affected by sample difference, tissue specificity, extracted RNA quality, and other factors, the expression analysis of genes requires normalization to reference genes [14]. Several studies have shown that the *β-actin* and *glyceraldehyde-3-phosphate dehydrogenase* (*GAPDH*) were selected as the RT-qPCR internal reference genes in *Rubus niveus* and *Rubus idaeus*, respectively [15,16]. However, studies on the reference genes of *Rubus* have not been systematically reported. In this study, 12 commonly used candidate reference genes were screened based on transcriptome data to study the internal reference genes of raspberry and blackberry in different organs and at different fruit developmental stages. The results showed that the *RuEEF1A* and *Ru18S* genes had the highest stability and were the most suitable reference genes of *Rubus*, laying the foundation for gene expression analysis and functional studies of blackberry and raspberry in the future.

The key to the standardization of gene expression levels is to screen suitable reference genes. An internal reference gene is a gene that is relatively stable in organisms regardless of changes in conditions or different tissues or parts [17,18]. Ideal reference genes are usually characterized by stable expression under different physiological conditions [18,19]. Unfortunately, the expression of each gene is affected by a variety of factors and varies under different experimental conditions or in different tissues [19,20]. The selection of reference genes is not universal. It is important to screen reference genes with relatively stable expression under specific experimental conditions [21]. Common reference genes may be involved in important components of the cytoskeleton (*TUA* and *TUB*) or in basic biochemical metabolic processes in organisms (*UBQ*) [22] and are relatively stable in corresponding tissues and organs [23,24]. Brunner et al. [25] analyzed the expression stability of 10 reference genes in different tissues of poplar and found that *UBQ* was the gene with the highest expression stability among all materials. The results of this study are different, and the stability of reference genes is different in different species. In addition, *18S* ribosomal RNA is the most abundant ribosomal RNA in eukaryotic organisms and is often used to analyze internal genes. This study concluded that the *18S* expression was relatively stable for blackberry and raspberry internal genes. As a component of chloroplast ribosomes, *30S* was preferred as a stable internal parameter at different developmental stages of different tissues and seeds of the oil crop *Plukenetia volubilis*, but its expression was most unstable during flower development [26]. As a component of the eukaryotic ribosomal subunit, the *40S* ribosomal RNA was the most stable gene in all samples of *Tuber melanosporum* during development [27]. The *30S* and *40S* genes in this study showed moderate stability and were not the most stable reference genes. Therefore, the expression stability of the same reference gene is different among different species.

GeNorm [28], BestKeeper [29], NormFinder, the ΔCt [30] approach, and the stability index [25], in addition to other mathematical methods, have been used to assess the stability of internal genes [31]. The GeNorm and NormFinder algorithms are similar in that geNorm selects a pair of optimal reference genes under different conditions, rather than a single gene [32], while NormFinder only selects a relatively appropriate reference gene [33]. In contrast to the former two, BestKeeper determines relatively stable genes by directly calculating Ct values [34]. Due to the analysis software programs having different screening principles and emphases, the internal reference genes will be different. Therefore, the stability of internal reference genes was evaluated by the comprehensive use of geNorm, BestKeeper, and NormFinder in this study. The ranking values of the 12 candidate internal reference genes with the three statistical algorithms were added, and the sum was used as a new variable, representing the comprehensive evaluation of gene expression stability. The smaller the sequence value, the higher the stability of gene expression. In the study of gene expression analysis, using a single internal reference gene for correction and standardization sometimes cannot meet the experimental requirements and ensure the accuracy of the experimental results. Therefore, to reduce various errors in the experiment, it is necessary to introduce two or more internal reference genes for standardized correction [12]. Through comprehensive evaluation combined with validation analysis, *RuEEF1A* and *Ru18S* were identified as relatively suitable reference genes. The *EEF1A* and *18S* genes have not previously been used as reference genes for raspberry and blackberry, which further illustrates the importance of this study. However, several studies have shown that *Ru18S* is not a suitable reference gene for *Stevia rebaudiana* [35] and *Zanthoxylum bungeanum* [36], again demonstrating that the reference genes are different in different species.

## 4. Material and Methods

### 4.1. Plant Materials

The yellow raspberry cultivar ‘Colde Summit,’ red raspberry cultivar ‘Heritage,’ black raspberry cultivar ‘Bristol,’ and blackberry cultivar ‘Chester’ were used as the experimental materials. The green fruits, immature fruits and mature fruits, leaves, stems, and stem apexes were collected and stored in dry ice immediately. After that, these materials were placed in an ultralow-temperature refrigerator at −80 °C until RNA extraction. There were 3 biological replicates per sample. Blackberry and raspberry plants were grown at the test base of Nanjing Lishui White Horse Industrial Park, Institute of Botany, Jiangsu Province and the Chinese Academy of Sciences. These Rubus plants grow in the Lishui area (Nanjing, Jiangsu, China) (119°09′ E, 31°35′ N) with mild and humid climate, sufficient light, and simultaneous rain and heat. The specific climatic conditions are an annual average temperature of 16.4 °C, annual average relative humidity of 76%, annual average precipitation of 1204.3 mm, annual average rainy days of 123 days, and annual average sunshine of 1980 h.

### 4.2. Primer Design for Candidate Reference Genes

According to the transcriptome sequencing data of blackberry (accession number: PRJNA680622), 12 genes were selected as candidate reference genes based on the CDS results of annotation information from the NCBI Nr database, namely, 18S ribosomal RNA (Ru18S), 30S ribosomal RNA (Ru30S), 40S ribosomal RNA (Ru40S), tubulin alpha chain-like protein (RuTUBA), elongation factor 1-alpha (RuEEF1A), elongation factor 1-beta (RuEEF1B), eukaryotic initiation factor 4A (RuEF4A), F-box family protein (RuF-box), polyubiquitin (RuUBC), ubiquitin (RuUBQ), phospholipase A (RuPA), and phosphoglycerate kinase (RuPGK). The primers used for RT-qPCR were designed using Oligo 6.0 software (Table 1). The annealing temperature was between 60 and 61 °C, and the primers were sent to Nanjing TSINGKE Biological Technology Co., Ltd., for synthesis.

### 4.3. Total RNA Extraction and cDNA Synthesis

RNA was extracted from different cultivars and tissues and at different fruit developmental stages from blackberry and raspberry according to the instructions of the Plant Total RNA Extraction Kit (Bioteke, Beijing, China). RNA quality was detected by 1.5% (*w*/*v*) agarose gel electrophoresis, and RNA concentration and purity were detected by a NanoDrop 2000 (Thermo, Waltham, USA). Total RNA (1 µg) from each sample was reverse-transcribed into cDNA using a PrimeScript RT kit (Takara, Dalian, China) according to the instructions. The obtained cDNA was diluted 5 times and stored in a −20 °C refrigerator for subsequent RT-qPCR experiments.

### 4.4. RT-qPCR Amplification

TB Green Premix Ex Taq II (Tli RNaseH Plus) (Takara, Dalian, China) was used for RT-qPCR. The system was 20 µL in total and contained 10 µL of TB Green Premix, 0.6 µL of upstream and downstream primers (10 µmol/L), 0.6 μL of Rox Reference Dye II, 1.5 μL of cDNA template, and ddH_2_O to the final volume. The ABI Viia 7 real-time PCR Platform was used. The reaction procedures were as follows: (i) holding stage: predenaturation at 95 °C for 30 s; (ii) PCR stage: 40 cycles were performed at 95 °C for 3 s and 60 °C for 30 s; (iii) melt curve stage (at the end of the amplification reaction, the product specificity was detected by the melt curve): 95 °C for 15 s, 60 °C for 60 s, and 95 °C for 15 s.

### 4.5. Data Processing and Analysis

The RT-qPCR data (all sample cycle thresholds, Ct values) of different cultivars, tissues, and fruit developmental stages of blackberry and raspberry were collated and summarized by Excel 2013 software. Under the precondition that the algorithm principles were strictly followed, the reference gene stability analysis software programs geNorm [28], BestKeeper [37], and NormFinder [29] were used to evaluate the expression stability of different reference genes in blackberry and raspberry. In brief, geNorm software was used to calculate the M value based on the pairwise variation between two reference genes, and the genes with the lowest expression stability were gradually eliminated. If the M value is less than 1.5, it can be considered as an internal reference gene. The smaller the M value, the higher the stability. GeNorm software determined the number of ideal candidate reference genes by calculating the V value of pairing variation. The default V value is 0.15; when Vn/n + 1 is less than 0.15, the number of internal reference genes of RT-qPCR should be n without introducing n + 1, to determine the optimal number of required internal reference genes [28]. NormFinder calculated the S value for reference genes according to variance analysis, and the smaller S is, the higher the stability [29]. BestKeeper evaluated the expression stability of all candidate reference genes by calculating the SD and CV of the original Ct values. When the SD is more than 1, the gene cannot be used as an internal reference gene. The lower the SD value, the lower the CV value, and the higher the R value, the more stable the expression of the candidate reference genes [37].

### 4.6. Validation of Selected Reference Genes

To verify the effect of the selection of different reference genes on the final standardization results, the relative expression level of the *RuCYP73A* gene in the different tissues and fruit developmental stages of blackberry was analyzed by using a single stably or unstably expressed gene or a combination of stable reference genes. The fold change in gene expression was calculated by the 2^−∆∆ct^ method.

## 5. Conclusions

We analyzed the expression levels of 12 candidate reference genes in four cultivars of *Rubus*, blackberry, black raspberry, red raspberry, and yellow raspberry, in different tissues and at different fruit developmental stages. Based on the comprehensive ranking analysis with three statistical algorithms, geNorm, NormFinder, and BestKeeper, it was found that there were differences in the optimal reference gene combinations among experimental groups, which indicated that none of the genes were suitable for use as a normalized reference gene under all the experimental conditions. The expression of the *RuEEF1A* and *Ru18S* genes in all the samples was the most relatively stable, and these were the most suitable thermostatic reference genes of blackberry and raspberry, laying a foundation for accurate gene expression analyses in the future. In summary, we recommend the use of at least two internal reference genes in further experiments.

## Figures and Tables

**Table 1:** Primer sequences of candidate reference genes.

Gene Name	Forward Primer (5′-3′)	Reverse Primer (5′-3′)	Product Length (bp)
*Ru* *18S*	ACGTCATCCTCCGGCAAAGC	ACGACGAAGCTCGCAAGTACAC	103
*Ru* *30S*	ACCCGACTTGCGTCCTACACT	AGCGCTTGACCCATTGGAAGC	119
*Ru* *40S*	GGGACCAAGCCATGGGTAAGC	CTAAGCTGCGGCTGTGGACTG	105
*Ru* *TUBA*	ATCCTTCTCGAGGGCGGCAA	AAGCGTGCGTTTGTGCACTG	93
*Ru* *EEF1A*	CCTTTCGCGCTCAGCCTTGA	AAGTCGACCACCACGGGTCA	150
*Ru* *EEF1B*	CCACCATGGCCGTCACCTTC	GACGAAACGCAGCCGTACCA	193
*Ru* *EF4A*	GTGCAGCAGGTCTCGCTTGT	TGGCAACACCCTTCCTCCCA	106
*Ru* *F-box*	GGGATCCATTGCCAGCAGCA	GCAGCCGGAGAAGGATGTCTG	72
*Ru* *UBC*	AGGGAATCCCACCGGACCAG	TCAGCCAAAGTGCGACCATCC	73
*Ru* *UBQ*	GGCCGCACCCTTGCAGATTA	TGCATCCCACCACGTAGACGA	77
*Ru* *PA*	GCTTCAGCAAGACTCCCATAAGGC	CAGGCAGAGCTCGTTGGTTGT	104
*Ru* *PGK*	ACTAGGGTCCGTGCTGCTGT	CAGCAAATCCACCACAACCCACA	191

**Table 2:** Stability analysis of 12 candidate reference genes based on BestKeeper software.

**Rank**	**All Samples**			**Three Raspberries**			**All Fruit Developmental Stages**			**Raspberry Fruit Developmental Stages**			**Red Raspberry Tissues**			**Black Raspberry Tissues**		
	**Gene Name**	**SD**	**CV (%)**	**Gene Name**	**SD**	**CV (%)**	**Gene Name**	**SD**	**CV (%)**	**Gene Name**	**SD**	**CV (%)**	**Gene Name**	**SD**	**CV (%)**	**Gene Name**	**SD**	**CV (%)**
1	*RuEEF1A*	0.13	0.52	*RuEEF1A*	0.14	0.56	*RuEEF1A*	0.11	0.45	*Ru18S*	0.13	0.55	*Ru18S*	0.14	0.59	*RuTUBA*	0.13	0.53
2	*Ru18S*	0.17	0.73	*Ru18S*	0.15	0.63	*Ru18S*	0.14	0.59	*RuEEF1A*	0.14	0.56	*RuTUBA*	0.16	0.62	*Ru18S*	0.14	0.58
3	*RuTUBA*	0.22	0.88	*RuTUBA*	0.24	0.96	*RuTUBA*	0.25	1.01	*RuEF4A*	0.22	0.87	*RuEEF1A*	0.17	0.66	*RuEEF1A*	0.18	0.73
4	*RuUBC*	0.28	1.16	*RuUBC*	0.24	1.02	*RuEF4A*	0.25	1.00	*RuTUBA*	0.29	1.15	*RuUBC*	0.24	1.00	*RuEF4A*	0.24	0.94
5	*RuEF4A*	0.3	1.18	*RuEF4A*	0.29	1.14	*RuUBC*	0.36	1.48	*RuUBC*	0.31	1.28	*RuEEF1B*	0.30	1.21	*RuUBC*	0.28	1.17
6	*RuEEF1B*	0.33	1.34	*RuEEF1B*	0.37	1.48	*RuEEF1B*	0.39	1.55	*RuEEF1B*	0.41	1.63	*RuEF4A*	0.31	1.25	*RuEEF1B*	0.29	1.16
7	*RuUBQ*	0.81	2.99	*RuUBQ*	0.84	3.08	*RuPA*	0.71	2.67	*RuF-box*	0.50	1.64	*Ru40S*	0.48	1.95	*Ru40S*	0.34	1.28
8	*Ru40S*	0.98	3.89	*Ru40S*	0.96	3.85	*RuUBQ*	0.89	3.25	*RuPA*	0.55	2.11	*RuUBQ*	0.52	1.95	*RuUBQ*	0.53	1.86
9	*RuPA*	1.15	4.35	*RuPA*	1.01	1.25	*Ru40S*	0.96	3.81	*RuUBQ*	0.87	3.18	*RuPGK*	0.73	3.06	*RuF-box*	0.81	2.71
10	*Ru30S*	1.21	4.91	*RuF-box*	1.05	3.54	*RuF-box*	0.98	3.26	*Ru40S*	1.09	4.32	*RuF-box*	0.76	2.52	*Ru30S*	0.83	3.31
11	*RuPGK*	1.23	4.86	*Ru30S*	1.19	4.85	*Ru30S*	1.17	4.68	*RuPGK*	1.17	4.68	*Ru30S*	1.06	4.34	*RuPA*	0.91	3.42
12	*RuF-box*	1.30	4.44	*RuPGK*	1.25	5.01	*RuPGK*	1.21	4.78	*Ru30S*	1.27	5.18	*RuPA*	1.09	4.25	*RuPGK*	1.23	4.97
**Rank**	**Yellow Raspberry Tissues**			**Blackberry Tissues**			**Red Raspberry Fruit Developmental Stages**			**Black Raspberry Fruit Developmental Stages**			**Yellow Raspberry Fruit Developmental Stages**			**Blackberry Fruit Developmental Stages**		
	**Gene Name**	**SD**	**CV (%)**	**Gene Name**	**SD**	**CV (%)**	**Gene Name**	**SD**	**CV (%)**	**Gene Name**	**SD**	**CV (%)**	**Gene Name**	**SD**	**CV (%)**	**Gene Name**	**SD**	**CV (%)**
1	*RuEEF1A*	0.07	0.28	*RuEEF1A*	0.06	0.25	*Ru18S*	0.14	0.60	*RuTUBA*	0.12	0.46	*Ru18S*	0.08	0.36	*RuEEF1A*	0.02	0.08
2	*Ru18S*	0.09	0.36	*RuTUBA*	0.09	0.36	*RuEF4A*	0.15	0.58	*Ru18S*	0.13	0.56	*RuEEF1A*	0.10	0.41	*RuTUBA*	0.04	0.18
3	*RuEEF1B*	0.18	0.72	*RuEEF1B*	0.17	0.70	*RuEEF1A*	0.15	0.61	*RuEEF1A*	0.15	0.59	*Ru40S*	0.10	0.43	*Ru18S*	0.16	0.70
4	*RuUBC*	0.19	0.79	*Ru18S*	0.17	0.71	*RuUBQ*	0.18	0.68	*RuEF4A*	0.18	0.69	*RuEF4A*	0.14	0.55	*RuEEF1B*	0.22	0.88
5	*RuTUBA*	0.25	1.01	*RuEF4A*	0.31	1.22	*RuTUBA*	0.19	0.77	*Ru40S*	0.19	0.71	*RuTUBA*	0.15	0.62	*RuEF4A*	0.33	1.29
6	*RuEF4A*	0.25	1.01	*RuUBC*	0.32	1.32	*RuUBC*	0.21	0.87	*RuUBQ*	0.25	0.86	*RuEEF1B*	0.22	0.89	*RuUBC*	0.47	1.98
7	*Ru40S*	0.26	1.11	*RuPGK*	0.57	2.17	*Ru30S*	0.24	0.96	*RuPA*	0.27	1.04	*RuF-box*	0.23	0.76	*Ru40S*	0.58	2.32
8	*RuUBQ*	0.38	1.41	*RuUBQ*	0.73	2.69	*RuEEF1B*	0.41	1.65	*RuEEF1B*	0.38	1.50	*RuUBC*	0.24	1.00	*RuPA*	0.66	2.42
9	*RuPA*	0.83	3.23	*RuF-box*	0.74	2.64	*RuF-box*	0.44	1.42	*RuUBC*	0.39	1.59	*RuUBQ*	0.37	1.40	*Ru30S*	0.69	2.68
10	*RuPGK*	0.90	3.44	*Ru40S*	0.97	3.81	*RuPGK*	0.50	2.07	*RuF-box*	0.58	1.94	*Ru30S*	0.57	2.49	*RuPGK*	0.73	2.79
11	*Ru30S*	1.19	5.00	*Ru30S*	1.04	4.14	*Ru40S*	0.51	2.04	*Ru30S*	0.76	2.98	*RuPA*	0.71	2.74	*RuUBQ*	0.81	2.92
12	*RuF-box*	1.49	5.11	*RuPA*	1.24	4.57	*RuPA*	0.61	2.29	*RuPGK*	1.35	5.45	*RuPGK*	1.33	5.16	*RuF-box*	1.08	3.80

**Table 3:** Comprehensive analysis and ranking of 12 candidate reference genes.

**Rank**	**All Samples**					**Three Raspberries**					**All Fruit Developmental Stages**					**Raspberry Fruit Developmental Stages**				
	**Gene**	**G**	**N**	**B**	**S**	**Gene**	**G**	**N**	**B**	**S**	**Gene**	**G**	**N**	**B**	**S**	**Gene**	**G**	**N**	**B**	**S**
1	*RuEEF1A*	1	2	1	4	*Ru18S*	1	3	2	6	*RuEEF1A*	1	3	1	5	*RuEF4A*	2	1	3	6
2	*Ru18S*	1	3	2	6	*RuEEF1A*	1	4	1	6	*Ru18S*	1	4	2	7	*Ru18S*	1	5	1	7
3	*RuTUBA*	2	1	3	6	*RuTUBA*	2	1	3	6	*RuEF4A*	2	1	4	7	*RuEEF1A*	1	4	2	7
4	*RuUBC*	4	4	4	12	*RuUBC*	3	2	4	9	*RuTUBA*	3	2	3	8	*RuTUBA*	3	2	4	9
5	*RuEF4A*	3	5	5	13	*RuEF4A*	4	6	5	15	*RuUBC*	4	5	5	14	*RuUBC*	4	3	5	12
6	*RuEEF1B*	5	6	6	17	*RuEEF1B*	5	5	6	16	*RuEEF1B*	5	6	6	17	*RuEEF1B*	5	6	6	17
7	*RuUBQ*	6	7	7	20	*RuUBQ*	6	7	7	20	*RuUBQ*	6	7	8	21	*RuF-box*	6	9	7	22
8	*Ru40S*	7	8	8	23	*Ru40S*	7	8	8	23	*Ru40S*	7	8	9	24	*RuPA*	7	7	8	22
9	*Ru30S*	8	9	10	27	*RuPA*	9	9	9	27	*RuPA*	8	9	7	24	*Ru30S*	10	11	12	23
10	*RuPA*	9	10	9	28	*Ru30S*	8	10	11	29	*Ru30S*	9	10	11	30	*RuPGK*	11	12	11	24
11	*RuF-box*	10	11	12	33	*RuF-box*	10	11	10	31	*RuF-box*	10	11	10	31	*RuUBQ*	8	8	9	25
12	*RuPGK*	11	12	11	34	*RuPGK*	11	12	12	35	*RuPGK*	11	12	12	35	*Ru40S*	9	10	10	29
**Rank**	**Red Raspberry Tissues**					**Black Raspberry Tissues**					**Yellow Raspberry Tissues**					**Blackberry Tissues**				
	**Gene**	**G**	**N**	**B**	**S**	**Gene**	**G**	**N**	**B**	**S**	**Gene**	**G**	**N**	**B**	**S**	**Gene**	**G**	**N**	**B**	**S**
1	*Ru18S*	2	3	1	6	*RuTUBA*	1	2	1	4	*RuEEF1A*	1	3	1	5	*RuEEF1A*	1	1	1	3
2	*RuUBC*	1	1	4	6	*RuEEF1A*	1	1	3	5	*RuUBC*	2	1	4	7	*RuTUBA*	1	3	2	6
3	*RuTUBA*	1	4	2	7	*Ru18S*	2	6	2	10	*Ru18S*	1	7	2	10	*Ru18S*	2	2	4	8
4	*RuEEF1A*	3	5	3	11	*RuEF4A*	3	5	4	12	*RuTUBA*	3	2	5	10	*RuEEF1B*	3	4	3	10
5	*RuEEF1B*	4	7	5	16	*RuUBC*	4	8	5	17	*RuEF4A*	4	4	6	14	*RuEF4A*	4	5	5	14
6	*RuUBQ*	6	2	8	16	*Ru40S*	6	4	7	17	*RuEEF1B*	6	6	3	15	*RuUBC*	5	6	6	17
7	*RuEF4A*	5	8	6	19	*RuEEF1B*	5	7	6	18	*RuUBQ*	7	8	8	15	*RuPGK*	6	8	7	21
8	*Ru40S*	7	6	7	20	*RuUBQ*	7	3	8	18	*Ru40S*	5	5	7	17	*RuUBQ*	7	7	8	22
9	*RuPGK*	8	10	9	27	*RuF-box*	8	9	9	26	*RuPA*	8	9	9	26	*RuF-box*	8	10	9	27
10	*RuF-box*	9	9	10	28	*Ru30S*	9	10	10	29	*RuPGK*	9	10	10	29	*Ru40S*	9	9	10	28
11	*Ru30S*	10	11	11	32	*RuPA*	10	11	11	32	*Ru30S*	10	11	11	32	*Ru30S*	10	11	11	32
12	*RuPA*	11	12	12	35	*RuPGK*	11	12	12	35	*RuF-box*	11	12	12	35	*RuPA*	11	12	12	35
**Rank**	**Red Raspberry Fruit Developmental Stages**					**Black Raspberry Fruit Developmental Stages**					**Yellow Raspberry Fruit Developmental Stages**					**Blackberry Fruit Developmental Stages**				
	**Gene**	**G**	**N**	**B**	**S**	**Gene**	**G**	**N**	**B**	**S**	**Gene**	**G**	**N**	**B**	**S**	**Gene**	**G**	**N**	**B**	**S**
1	*RuEF4A*	1	1	2	4	*Ru18S*	3	1	2	6	*Ru18S*	1	4	1	6	*RuTUBA*	1	1	2	4
2	*RuEEF1A*	1	2	3	6	*RuTUBA*	1	4	1	6	*RuEEF1A*	1	5	2	8	*RuEEF1A*	1	2	1	4
3	*RuUBQ*	3	3	4	10	*RuEEF1A*	2	2	3	7	*Ru40S*	2	3	3	8	*Ru18S*	2	3	3	8
4	*Ru18S*	5	5	1	11	*Ru40S*	1	5	5	11	*RuEF4A*	3	1	4	8	*RuEEF1B*	3	4	4	11
5	*RuUBC*	2	4	6	12	*RuEF4A*	4	3	4	11	*RuTUBA*	4	2	5	11	*RuEF4A*	4	5	5	14
6	*RuTUBA*	4	6	5	15	*RuUBQ*	5	6	6	17	*RuEEF1B*	5	6	6	17	*RuUBC*	5	7	6	18
7	*Ru30S*	6	7	7	20	*RuPA*	6	7	7	20	*RuF-box*	6	9	7	22	*Ru40S*	8	6	7	21
8	*RuEEF1B*	7	11	8	26	*RuEEF1B*	7	8	8	23	*RuUBC*	7	7	8	22	*RuPA*	6	10	8	24
9	*Ru40S*	8	8	11	27	*RuUBC*	8	9	9	26	*RuUBQ*	8	8	9	25	*Ru30S*	7	11	9	27
10	*RuF-box*	9	10	9	28	*RuF-box*	9	10	10	29	*Ru30S*	9	10	10	29	*RuPGK*	9	8	10	27
11	*RuPGK*	10	9	10	29	*Ru30S*	10	11	11	32	*RuPA*	10	11	11	32	*RuUBQ*	10	9	11	30
12	*RuPA*	11	12	12	35	*RuPGK*	11	12	12	35	*RuPGK*	11	12	12	35	*RuF-box*	11	12	12	35

G: gene expression stability ranking in geNorm. N: gene expression stability ranking in NormFinder. B: gene expression stability ranking in BestKeeper. S: the sum of gene stability rankings in the three algorithms.

## Data Availability

The data and materials supporting the conclusions of this study are included within the article.
